# Sleep Bruxism and Occlusal Function: A Case–Control Study Based on Polysomnography in Young Colombians

**DOI:** 10.3390/jcm14196733

**Published:** 2025-09-24

**Authors:** Juan Alberto Aristizabal-Hoyos, Olga López-Soto, Héctor Fuentes-Barría, Raúl Aguilera-Eguía, Lissé Angarita-Davila, Diana Rojas-Gómez

**Affiliations:** 1 Departamento de Salud Oral, Facultad de Salud, Universidad Autónoma de Manizales, Caldas 170008, Colombia; jaristi@autonoma.edu.co (J.A.A.-H.); sonrie@autonoma.edu.co (O.L.-S.); 2 Vicerrectoría de Investigación e Innovación, Universidad Arturo Prat, Iquique 1110939, Chile; 3 Departamento de Salud Pública, Facultad de Medicina, Universidad Católica de la Santísima Concepción, Concepción 3349001, Chile; raguilerae@ucsc.cl; 4 Escuela de Nutrición y Dietética, Facultad de Medicina, Universidad Andres Bello, Concepción 3349001, Chile; lisse.angarita@unab.cl; 5 Escuela de Nutrición y Dietética, Facultad de Medicina, Universidad Andres Bello, Santiago 7550000, Chile; diana.rojas@unab.cl

**Keywords:** sleep bruxism, dental occlusion, temporomandibular joint, tooth wear, physical examination, adult, case–control studies

## Abstract

**Objectives**: The objective of this study was to compare occlusal and articular characteristics between young adults with and without a confirmed diagnosis of sleep bruxism, through a case-control study based on polysomnography. **Methods**: We conducted a case–control study with probabilistic sampling, including 20 participants with a polysomnography-confirmed diagnosis of sleep bruxism and 20 age- and sex-matched controls. A clinical oral examination was conducted to identify wear facets, joint sounds, and soft tissue indentations. Occlusal relationships were analyzed using mounted models on a semi-adjustable articulator, evaluating interferences during working, balancing, and protrusive movements, premature contacts, attrition, and dental inclinations. **Results**: Sleep bruxism showed a higher frequency of temporomandibular joint sounds (35% vs. 5%; *p* = 0.017; w = 0.375) and left-side balancing interferences (*p* = 0.04; d = 0.723). Multivariate analysis revealed a moderate correlation between bruxism and the combination of joint sound and occlusal inclination (COR = 0.39; 95% CI: 0.19–0.57; I^2^ = 0.0%). Joint sound showed a weak association with REM-related bruxism (COR = 0.29; 95% CI: 0.05–0.51; I^2^ = 21.7%) and a moderate association with non-REM bruxism (COR = 0.41). The correlation with occlusal inclination was stronger during REM sleep (COR = 0.41) than during non-REM sleep (COR = 0.35; I^2^ = 0.0%), indicating consistent and clinically relevant associations. **Conclusions**: Occlusal and functional characteristics associated with sleep bruxism were identified, particularly joint sounds and dental inclinations, although no direct causal relationship was established. These findings suggest the presence of specific morphofunctional patterns that may play a role in the clinical expression of sleep bruxism.

## 1. Introduction

Sleep bruxism is defined as a rhythmic, involuntary, and repetitive mandibular activity that occurs during sleep, typically accompanied by grinding sounds [1,2]. It is currently understood as a complex, multisystemic physiological phenomenon with a multifactorial etiology, moving beyond the traditional mechanical paradigm [1,2,3,4].

This conceptual shift has redirected research toward the potential effects of bruxism on the stomatognathic system, particularly its role in the development of clinical dysfunctions that may require therapeutic intervention [4]. Several studies have reported associations between sleep bruxism, temporomandibular disorders (TMDs), and postural abnormalities [5,6]. This parafunctional activity can occur both during wakefulness and sleep, manifesting as involuntary clenching or grinding patterns [7,8,9].

For decades, various theories attributed bruxism to local factors, such as occlusal interferences or anatomical characteristics of the orofacial complex. However, these hypotheses have been increasingly questioned because of their limited empirical support and marginal clinical relevance [8,10]. In parallel, diverse etiological factors have been proposed, including systemic conditions (e.g., intestinal parasitosis, nutritional deficiencies, allergies, or endocrine disorders), psychological variables, and local factors such as malocclusion [3,8,11,12]. Specifically, the interaction of emotional stress, orofacial pain, and occlusal discrepancies has been suggested to promote the onset of bruxism [11,12,13]. More recent research, however, emphasizes the predominant role of psychopathological and neurophysiological mechanisms, relegating morphological factors to a secondary position [8,14]. While malocclusion has traditionally been considered a potential causal factor, there is no conclusive evidence to support this relationship [5,6,13]. Consequently, current approaches prioritize psychological, biological, and exogenous determinants, in alignment with the multifactorial nature of bruxism [3,8,14,15].

Today, sleep bruxism is recognized as a condition primarily of central origin, even though its most evident clinical manifestations appear within the stomatognathic system [8,16]. This paradigm shift—from an occlusion-centered to a neurophysiological model—has been supported by findings linking sleep bruxism to micro-arousals and alterations in D2 dopamine receptor expression in the striatum of affected individuals [17]. Its prevalence ranges between 20% and 40%, depending on the subtype (nonspecific, sleep-related, or awake bruxism), the diagnostic method used (self-report, clinical evaluation, or polysomnography), and population characteristics. It is especially prevalent among young adults; therefore, in the context of this study, diagnosis based on polysomnography was estimated at 43% [3,18].

Given its high prevalence and its potential association with functional disturbances in the stomatognathic system, it is relevant to explore its relationship with occlusal parameters. In this context, the present study aimed to compare occlusal and articular parameters in young adults with and without a confirmed diagnosis of sleep bruxism through a retrospective case–control study.

## 2. Materials and Methods

### 2.1. Design

This case–control study was conducted in accordance with the STROBE guidelines for observational research (Supplementary Material) [19]. The research protocol was approved by the Ethics Committee of the Autonomous University of Manizales (Protocol Code: GIN–GUN–001) and complied with Colombian Ministry of Health Resolution No. 8430 of 1993 and the ethical principles of the Declaration of Helsinki [20].

### 2.2. Context

This study was carried out in 2016 as part of the Oral Health Prevention Program at the Autonomous University of Manizales (Colombia). The target population included male and female university students aged 18 to 35 years.

The diagnosis of sleep bruxism was confirmed by nocturnal polysomnography (PSG), the gold standard for detecting rhythmic mandibular activity during sleep. This was complemented by a functional and morphological analysis of dental casts mounted on a semi-adjustable articulator, enabling detailed evaluation of both static and dynamic occlusal parameters, as well as associated clinical signs.

Participants were selected through probabilistic sampling, including only those with complete clinical records and valid diagnostic documentation. All participants were informed about the study objectives, selection criteria, and the retrospective nature of the research. Informed consent was obtained, authorizing the scientific use of their data for a period of up to ten years.

### 2.3. Participants

The final sample consisted of 40 university students, equally divided into two groups:

- Case group: 20 participants with confirmed sleep bruxism diagnosis via PSG.
- Control group: 20 participants without bruxism, matched by age and sex.

All participants were enrolled in the Oral Health Prevention Program and had complete clinical records, valid dental assessments, and PSG results.

Inclusion criteria: Students aged 18 to 35, actively enrolled in 2016, with complete clinical documentation including dental and PSG evaluations.

Exclusion criteria: Prior diagnosis of neuromuscular disorders or craniofacial malformations, ongoing orthodontic or prosthetic treatment, history of orthognathic surgery during the evaluation period, or technical inability to obtain functional articulator models.

### 2.4. Sleep Bruxism

Diagnosis followed a three-step protocol with increasing sensitivity and specificity, based on established guidelines (Supplementary Material) [21]:

Self-reported symptoms: Considered positive if the participant or a third party reported grinding sounds during sleep, accompanied by temporal or masseter muscle pain/fatigue upon awakening.

Intraoral clinical evaluation: Conducted by a certified oral rehabilitation specialist, screening for the following:

- Atypical dental wear facets (anterior or posterior).
- Masseter muscle hypertrophy during voluntary clenching.
- Morning muscle pain, discomfort, or fatigue not attributable to other causes.

Polysomnography: Positive cases in the first two levels were referred to the university laboratory, where a PSG was performed, recognized as the gold standard for identifying rhythmic mandibular activity and muscular events associated with sleep bruxism [22,23]. A physiatry specialist trained in PSG interpretation, blinded to participants’ clinical profiles, used a Cadwell Easy III system with 10 mm stainless steel disc electrodes (Cadwell^®^ 302139-200), following American Academy of Sleep Medicine (AASM) diagnostic criteria [24].

Electrodes were placed on the scalp using conductive gel after skin preparation to maintain impedance below 5 kΩ throughout the night. Neuroelectric activity was recorded with standard anatomical references using the 10–20 international system, and signals were amplified and digitized at ≥500 Hz, with 12–16-bit resolution [23,24].

Eye movements were tracked via electrooculography, placing electrodes on the outer canthi (1 cm above for the right eye, 1 cm below for the left), referenced to a mastoid electrode. This configuration enabled detection of vertical and horizontal eye movements through opposite-polarity signals, facilitating REM detection and transitions from wakefulness to light sleep. Slow eye movements were defined as those with <500 ms latency from initial deflection to peak amplitude [23,24].

Only cases confirmed through PSG were classified as sleep bruxism. For each positive case, an age- and sex-matched control was randomly selected from eligible candidates.

### 2.5. Functional Occlusal Assessment

Maxillary and mandibular models were mounted on a semi-adjustable articulator to analyze static and dynamic interocclusal relationships [25], following previous guidelines [26,27,28]. The assessment included the following:

- Premature contacts.
- Wear facets (attrition, abrasion, erosion).
- Occlusal interferences during work, balancing, and protrusive movements.

Dental morphology included Spee and Wilson curves, arch and palatal shape, gingival contours, malpositioned teeth, and the number of functionally absent units.

Temporomandibular joints were also examined for joint sounds, functional blockages, and muscle hypertrophy. Indirect signs of parafunction (e.g., cheek or tongue mucosa indentations) were noted as well [29].

### 2.6. Bias

As a case–control design, this study acknowledged potential biases [30].

- Selection bias may have arisen from the inclusion of only students with complete records and valid bruxism diagnostics, potentially limiting generalizability. This was mitigated through probabilistic sampling and strict inclusion criteria.
- Information bias, especially regarding self-reported symptoms, was minimized by employing a hierarchical, three-stage diagnostic protocol (self-report, clinical inspection, PSG), enhancing diagnostic accuracy.
- Observer bias was addressed by ensuring that PSG evaluations and clinical interpretations were conducted by blind professionals, using standardized and validated protocols.

### 2.7. Sample Size

The sample size was calculated based on the university’s student population, aged 18–35, enrolled in the Oral Health Prevention Program in 2016. Using Epi Info v7 (StatCalc), and assuming 60% exposure prevalence in the bruxism group and 10% in controls, with a 1:1 case–control ratio, an expected odds ratio of 2.0, a 95% confidence interval, and 80% power, the required sample size was 40 participants. The 60% prevalence in the bruxism group is based on a previous study that documented a high prevalence of sleep bruxism in specific groups, such as young adults and individuals with sleep disorders, which show prevalence rates of up to 60% [31,32].

Thus, the final sample comprised 20 confirmed bruxism cases and 20 matched controls.

### 2.8. Statistical Analysis

Data analysis was conducted using SPSS v27 (IBM Corp., Armonk, NY, USA). Categorical variables were expressed as absolute and relative frequencies and compared using Pearson’s chi-square test.

The normality of continuous variables was assessed with the Shapiro–Wilk test. For normally distributed variables, independent-samples t-tests were applied, reporting means, standard deviations (SDs), and standard errors (SEs). Odds ratios (ORs) with 95% confidence intervals (CIs) were also calculated.

The effect size was determined based on data type: Cramér’s V (w) for categorical variables (interpreted as small < 0.1, moderate 0.1–0.3, large ≥ 0.3), and Cohen’s d (d) for continuous variables (small ≥ 0.1, moderate ≥0.3, large ≥ 0.7) [33,34]. These metrics allowed the identification of clinically relevant differences, even in the absence of statistical significance.

To explore multivariable associations between sleep bruxism, occlusal conditions, and TMD symptoms, three linear regression models were developed. Model 0 included the total number of bruxism events as the dependent variable; Model 1 adjusted for the presence of joint sounds; and Model 2 additionally incorporated dynamic occlusal features (e.g., interferences, tooth inclination). Correlation coefficients (COR) with 95% confidence intervals (CIs) were reported, and a two-tailed significance level of *p* < 0.05 was applied.

All regression analyses were conducted under a random effects model to account for potential variability between subjects beyond sampling error. To evaluate heterogeneity, the I^2^ statistic proposed by Higgins and Thompson was calculated. This measure estimates the proportion of total variability in effect sizes that cannot be attributed to sampling error (i.e., intra-group variability). I^2^ values were interpreted using conventional thresholds: values below 25% indicated low heterogeneity, values below 50% indicated moderate heterogeneity, and values below 75% indicated substantial heterogeneity. In addition, T^2^ (tau-squared) was computed to estimate the between-subject variance, providing a complementary indicator of unexplained heterogeneity in the models.

## 3. Results

The final sample consisted of 40 young adults, evenly divided between a sleep bruxism group (n = 20) and a control group (n = 20). The mean age was significantly higher in the sleep bruxism group (22.85 ± 3.67 years) compared to the control group (20.35 ± 1.57 years), with a statistically significant difference (*p* = 0.009) and a large effect size (d = 0.88). Regarding the sex distribution, no significant differences were found between the groups (*p* = 0.705), with a small effect size (w = 0.06). Other baseline variables, including smoking status, frequency of coffee consumption, self-reported stress, and the use of anxiolytic or sleep medication, showed no statistically significant differences between groups, with small to moderate effect sizes. No dropouts occurred during the course of this study (Table 1).

### 3.1. Comparison of Signs, Symptoms, and Medical History

Table 2 presents a comparison of clinical signs, symptoms, and history between subjects with and without sleep bruxism. Although most of the analyzed variables did not show statistically significant differences between the groups (*p* > 0.05), moderate effect sizes were observed for variables such as nocturnal clenching (w = 0.209), dental hypersensitivity (w = 0.154), the presence of occlusal impressions on buccal mucosa (w = 0.100), and temporomandibular joint (TMJ) locking (w = 0.284). Notably, the presence of joint sounds was significantly higher in the sleep bruxism group (35% vs. 5%), with a statistically significant difference (*p* = 0.017) and a large effect size (w = 0.375). This suggests a possible association between sleep bruxism and functional alterations of the TMJ.

### 3.2. Dynamic Occlusal Evaluation

Table 3 presents the results of the dynamic articulator analysis, evaluating occlusal interferences and functional parameters during mandibular excursive movements. In general, no statistically significant differences were observed between the bruxism and control groups regarding right or left lateral interferences, protrusion, horizontal or vertical overbite, or premature contacts. However, a significant difference was found in balancing interferences on the left side (*p* = 0.04), being more pronounced in the bruxism group. This variable not only showed statistical significance but also had a large effect size (d = 0.723), suggesting a clinically relevant association between sleep bruxism and balancing interferences. Moderate to large effect sizes were also observed in other variables, such as right balancing interferences (d = 0.556), vertical overbite (d = 0.829), and premature contacts (d = 2.154), although these did not reach statistical significance. These findings may reflect subtle functional misalignments in bruxism subjects that warrant further clinical and biomechanical exploration.

### 3.3. Correlational Analysis

**Figure 1:**
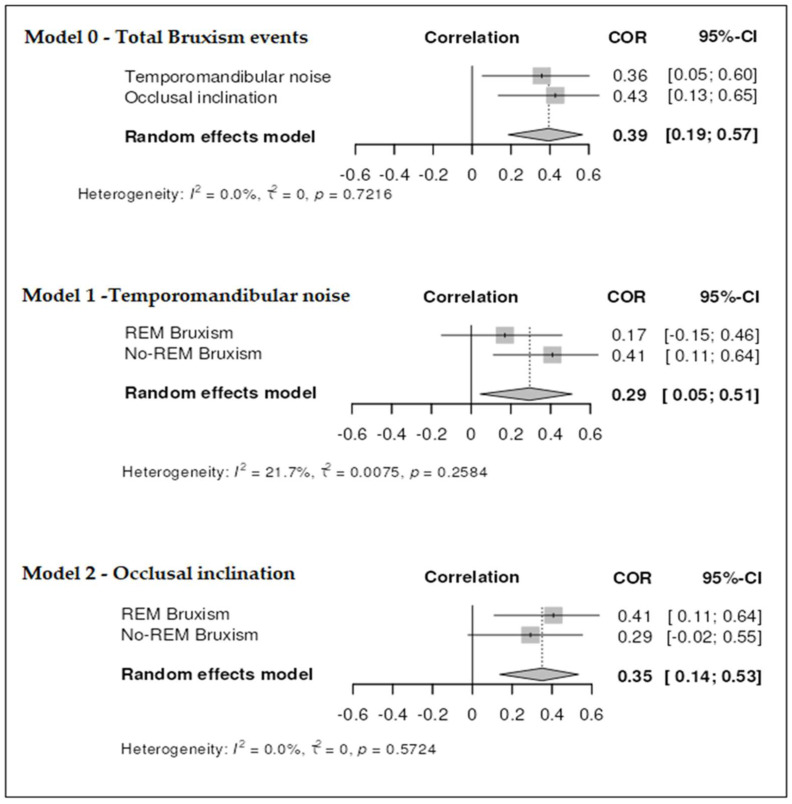
Multivariate associations between sleep bruxism, occlusal conditions, and temporomandibular symptoms.

Figure 1 presents the results of the correlation analysis among the studied variables using random effects models. In Model 0, a moderate and statistically significant correlation was observed between the total number of bruxism events and two occlusal–functional parameters: TMJ noises and occlusal inclination (COR = 0.39; 95% CI: 0.19–0.57). Heterogeneity was absent (I^2^ = 0.0%, τ^2^ = 0, *p* = 0.7216), indicating consistent effects across both variables.

In Model 1, a moderate correlation was found between TMJ noises and bruxism activity during REM and non-REM sleep phases (COR = 0.29; 95% CI: 0.05–0.51), with low and non-significant heterogeneity (I^2^ = 21.7%, τ^2^ = 0.0075, *p* = 0.2584), suggesting a slightly variable but still coherent association.

Model 2 revealed a moderate correlation between REM and non-REM bruxism phases and occlusal inclination (COR = 0.35; 95% CI: 0.14–0.53), with no heterogeneity observed (I^2^ = 0.0%, τ^2^ = 0, *p* = 0.5724), supporting the consistency of this relationship.

Overall, the absence or minimal presence of heterogeneity across models reinforces the robustness of the associations found.

## 4. Discussion

The main objective of this research was to compare occlusal and joint characteristics between individuals with a confirmed diagnosis of sleep bruxism and a matched control group, using a retrospective case–control design. One of the most relevant findings was the higher frequency of joint noises in the sleep bruxism group, a statistically significant difference supported by multivariate analysis, suggesting a possible functional association between this nocturnal parafunction and TMJ alterations. Previous studies have indicated that sleep bruxism may be related to repetitive microtrauma to the stomatognathic system, including elongation of capsular and discal ligaments, disc thinning, and muscular discoordination, which may contribute to condyle-disc displacement and the emergence of joint sounds [35,36]. This evidence supports the hypothesis that the TMJ is one of the structures most susceptible to the mechanical load associated with moderate to severe bruxism [37]. A significant association between bruxism and articular clicking has also been documented, especially when diagnosed through electromyography [38,39]. However, this relationship remains debated. Some authors suggest that bruxism patients may have greater subjective awareness of their symptoms, potentially overestimating the clinical relevance of this relationship, given that objective diagnostic methods tend to show less consistent correlations [40,41]. In this context, symptoms such as pain or joint sounds could reflect secondary muscular hyperactivity rather than true structural joint dysfunction. It has also been suggested that small changes in mandibular position could induce increased muscular activity and atypical mechanical loads on the TMJ, promoting inflammatory processes in retrodiscal or synovial tissues. Clinically, this may be present as joint sounds or disc dislocations in the presence of asymmetric occlusal contacts [42,43,44]. In this study, such findings were consistent with a higher frequency of joint sounds in the bruxism group, though without accompanying pain, possibly reflecting functional adaptation to chronic overload. However, it is crucial to highlight that despite the sample size calculation presented, several analyses yielded borderline results, which could indicate an insufficient sample size. Future studies should consider larger and more diverse samples to obtain more robust results. Additionally, it is important to emphasize that the inherent limitations of the obtained data, such as the absence of social variables like lifestyle, stress, and anxiety, among other comorbidities, were not addressed. These factors could have interfered with the observed results. Previous studies have reported that only a fraction of individuals with bruxism develop clinically significant orofacial pain [4,45,46].

Regarding occlusal variables, a higher frequency of interferences on the left balancing side was identified in individuals with sleep bruxism. This result aligns with findings by Tago et al. [47], who observed mediotrusive interferences in 96% of bruxism patients. However, despite this correlation, the correlation analysis performed in this study showed that while occlusal interferences have a moderate relationship with bruxism, the results may not have been sufficiently significant in all cases. The lack of a larger sample size could have influenced the borderline results observed. Additionally, the clinical relevance of these interferences remains controversial. Some studies suggest their contribution to the electromyographic pattern is limited and that their therapeutic removal may not be clinically justified [48]. Additionally, heterogeneity in the definition and classification of occlusal interferences complicates the establishment of consistent associations [38,49,50,51]. In contrast, no significant differences were observed between the groups regarding wearing facets, premature contacts, or working side interferences. These findings suggest caution when generalizing, as not all bruxism patients exhibit detectable occlusal alterations, and their presence does not necessarily imply a causal relationship. This aligns with studies that recommend a conservative approach to occlusal adjustments, reserving them for cases with clear clinical evidence of trauma, preferably combined with the use of occlusal splints [1,52,53]. A higher frequency of inclined teeth was also reported in the sleep bruxism group, which is consistent with the literature suggesting that dental inclinations may alter occlusal load trajectories and increase functional load on periodontal and articular structures [54,55]. These altered trajectories may also be associated with excessive attrition, headaches, and joint noises [29,56,57]. It is important to note that although the correlation is significant, the results may not be conclusive because of the limitations of the sample size and the lack of consideration of other social variables that may have influenced the severity of bruxism.

An unexpected finding was the lower dental attrition in the sleep bruxism group. This observation is consistent with reports questioning the validity of dental wear as a diagnostic marker for bruxism, as no consistent relationship has been demonstrated between attrition and the intensity or frequency of this parafunction [58,59]. However, it should be highlighted that this study’s limitations, especially regarding the sample size and the absence of factors such as stress and anxiety, may have influenced the results obtained. Overall, the results of this study support the hypothesis that sleep bruxism does not produce a uniform occlusal pattern. This variability may explain the lack of consensus regarding the intensity, clinical characteristics, and functional relevance of sleep bruxism, which seems to range between a physiological adaptive response and a pathological parafunction, even in the absence of evident articular or muscular manifestations [2,4,60].

## 5. Clinical and Practical Implications

The findings of this study provide relevant information for dental clinical practice, especially regarding the comprehensive diagnosis and functional assessment of sleep bruxism. The observed association between this parafunction and joint noises reinforces the usefulness of systematically including functional evaluation of the TMJ in patients presenting clinical signs of nocturnal parafunctional activity. The detection of occlusal interferences during balancing movements and dental inclinations should also be considered during clinical examinations, although these should not be assumed to indicate direct causality. In this context, a prudent and individualized clinical approach is recommended, avoiding invasive interventions, such as occlusal adjustments, unless clear evidence of dysfunction or trauma is present. Additionally, these findings may suggest a potential preventive role for orthodontics in the early correction of occlusal alterations that may increase functional load in predisposed patients. Overall, the need for interdisciplinary protocols is reinforced, integrating occlusal, functional, and psychosocial aspects to differentiate between physiological adaptations and pathological manifestations of sleep bruxism.

## 6. Study Limitations and Strengths

The case–control design limits the ability to establish causal relationships, as it does not allow for precise determination of the temporal sequence between occlusal characteristics and the presence of sleep bruxism. It is possible that some of the observed variables, such as balancing side interferences or dental inclinations, may function both as predisposing factors and as adaptive responses to parafunctional activity. These hypotheses warrant investigation in future longitudinal studies with clinical follow-up.

Additionally, the assessment of certain occlusal variables was based on subjective clinical criteria, which could introduce interobserver variability. However, standardized calibration procedures were implemented to minimize this potential bias and ensure consistency in data collection.

Despite these limitations, this study has several notable strengths. First, the sample was homogeneously distributed in terms of key sociodemographic factors, and no dropouts occurred. Second, the use of validated diagnostic criteria for sleep bruxism, combined with a structured occlusal–functional examination, enhances the internal validity of the findings. Third, the statistical analysis incorporated effect size measures and heterogeneity indices, which strengthen the interpretation of associations and the reliability of the results. Finally, the absence or minimal presence of heterogeneity across the correlation models supports the consistency and robustness of the identified relationships.

## 7. Conclusions

This study identified morphofunctional differences between young adults with and without a confirmed diagnosis of sleep bruxism. In particular, participants with bruxism showed a higher frequency of joint noises, occlusal interferences on the balancing side, and abnormal dental inclinations. Additionally, statistical associations were found between the number of bruxism events recorded via polysomnography and two specific features: TMJ noises and dental inclination. These findings support the hypothesis that sleep bruxism is related to specific, albeit heterogeneous, occlusal patterns that may reflect adaptive functional mechanisms or biomechanical decompensations. However, because of the retrospective design of this study, causal relationships cannot be established. Longitudinal research is needed to clarify the directionality and clinical relevance of these associations for a better understanding of sleep bruxism and its impact on stomatognathic function.

## Figures and Tables

**Table 1:** Baseline characteristics of the analyzed sample (n = 40).

Sociodemographic Variables	Sleep Bruxism Group (n = 20)	Control Group (n = 20)	*p*-Value	Effect Size Classification	
Age (years)	22.85 ± 3.67	20.35 ± 1.57	0.009	0.88 (d)	Large
Male	5 (25%)	4 (20%)	0.705	0.06 (w)	Small
Female	15 (75%)	16 (80%)			
Daily smoker	0 (0%)	1 (5%)	0.310	0.95 (w)	Large
Coffee >4 times/week	0 (0%)	2 (10%)	0.146	0.90 (w)	Large
Self-reported stress	8 (40%)	6 (30%)	0.507	0.10 (w)	Moderate
Anxiety medication	1 (5%)	3 (15%)	0.292	0.17 (w)	Moderate
Sleep medication	1 (5%)	2 (10%)	0.548	0.09 (w)	Small

d: effect size determined by Cohen’s d, w: effect size determined by Cramer’s V.

**Table 2:** Comparison of signs, clinical symptoms, and history between subjects with and without sleep bruxism (n = 40).

Signs, Clinical Symptoms, and History	Presents Condition	Sleep Bruxism Group	Control Group	*p*-Value	df	w	Effect Size Classification
Fatigue or sore jaw	Yes	16 (80%)	15 (75%)	0.708	1	0.059	Small
	No	4 (20%)	5 (25%)				
Sore teeth or gums	Yes	9 (45%)	7 (46.15%)	0.516	1	0.102	Moderate
	No	11 (55%)	13 (53.85%)				
Headache in the temporal region	Yes	11 (55%)	12 (60%)	0.751	1	0.050	Small
	No	9 (45%)	8 (40%)				
Daytime grinding	Yes	7 (35%)	6 (30%)	0.740	1	0.052	Small
	No	13 (65%)	14 (70%)				
Nighttime grinding	Yes	8 (40%)	5 (25%)	0.310	1	0.160	Moderate
	No	12 (60%)	15 (75%)				
Nighttime clenching	Yes	9 (45%)	5 (25%)	0.184	1	0.209	Moderate
	No	11 (55%)	15 (75%)				
Wear facets	Yes	14 (70%)	15 (75%)	0.718	1	0.057	Small
	No	6 (30%)	5 (25%)				
Masseter voluntary hypertrophy contraction	Yes	8 (40%)	10 (50%)	0.527	1	0.101	Moderate
	No	12 (60%)	10 (50%)				
Discomfort in masticatory muscles	Yes	12 (60%)	12 (60%)	1	1	0	Null
	No	8 (40%)	8 (40%)				
Dental hypersensitivity	Yes	9 (45%)	6 (30%)	0.327	1	0.154	Moderate
	No	11 (55%)	14 (70%)				
Temporomandibular joint locking	Yes	0 (0%)	3 (15%)	0.071	1	0.284	Moderate
	No	20 (100%)	17 (85%)				
Occlusal impressions on buccal mucosa	Yes	11 (55%)	9 (45%)	0.527	1	0.100	Moderate
	No	9 (45%)	11 (55%)				
Dental impressions on lingual edges	Yes	4 (20%)	7 (35%)	0.287	1	0.168	Moderate
	No	16 (80%)	13 (65%)				
Temporomandibular pain on palpation	Yes	2 (10%)	4 (20%)	0.377	1	0.139	Moderate
	No	18 (90%)	16 (80%)				
Temporomandibular pain when opening or closing the mouth	Yes	4 (20%)	2 (10%)	0.377	1	0.139	Moderate
	No	16 (80%)	18 (90%)				
Presence of joint noise	Yes	7 (35%)	1 (5%)	0.017	1	0.375	Large
	No	13 (65%)	19 (95%)				
Presence of functional anterior guidance	Yes	16 (80%)	16 (80%)	1	1	0	Null
	No	4 (20%)	4 (20%)				
Presence of premature contacts	Yes	19 (95%)	17 (85%)	0.292	1	0.166	Moderate
	No	1 (5%)	3 (15%)				

**Table 3:** Summary of dynamic occlusal analysis by groups (n = 40).

Dynamic Variables	Groups	Statisticians					
		SD	SE	95% IC	*p*-Value	d	Effect Size Classification
Right lateral interferences	Bruxism	0.74	0.16	0.65 (0.30 a 0.99)	0.53	0.128	Small
	Control	0.82	0.18	0.55 (0.16 a 0.93)			
Left lateral interferences	Bruxism	0.75	0.16	0.45 (0.09 a 0.80)	0.22	0.374	Moderate
	Control	0.85	0.19	0.75 (0.35 a 1.14)			
Right balancing interferences	Bruxism	0.58	0.13	0.31 (0.03 a 0.59)	0.07	0.556	Moderate
	Control	0.91	0.20	0.75 (0.32 a 1.17)			
Left balancing interferences	Bruxism	0.75	0.16	0.45 (0.09 a 0.80)	0.04	0.723	Large
	Control	0.22	0.05	0.05 (−0.05 a 0.16)			
Protrusion interferences	Bruxism	0.97	0.21	0.70 (0.24 a 1.16)	0.98	0.085	Null
	Control	1.13	0.26	0.79 (0.24 a 1.34)			
Horizontal overbite (mm)	Bruxism	0.68	0.15	1.50 (1.17 a 1.82)	0.67	0.092	Null
	Control	0.83	0.19	1.57 (1.17 a 1.98)			
Vertical overbite (mm)	Bruxism	0.67	0.15	1.65 (1.33 a 1.96)	0.21	0.829	Large
	Control	0.84	0.19	1.04 (1.53 a 2.35)			
Premature contacts	Bruxism	0.32	0.07	1.00 (0.84 a 1.15)	0.48	2.154	Large
	Control	0.52	0.12	0.94 (0.69 a 1.20)			

## Data Availability

The data from this article will be made available by the authors on reasonable request.

## Supplementary Materials

The following supporting information can be downloaded at https://www.mdpi.com/article/10.3390/jcm14196733/s1, S1: STROBE checklist, bruxism self-report instrument, and intraoral clinical evaluation.

## References

[1] Minakuchi H., Fujisawa M., Abe Y., Iida T., Oki K., Okura K., Tanabe N., Nishiyama A. (2022). Managements of sleep bruxism in adult: A systematic review. JPN. Dent. Sci. Rev..

[2] Verhoeff M.C., Lobbezoo F., Ahlberg J., Bender S., Bracci A., Colonna A., Dal Fabbro C., Durham J., Glaros A.G., Häggman-Henrikson B. (2025). Updating the Bruxism Definitions: Report of an International Consensus Meeting. J. Oral Rehabil.

[3] Bulanda S., Ilczuk-Rypuła D., Nitecka-Buchta A., Nowak Z., Baron S., Postek-Stefańska L. (2021). Sleep Bruxism in Children: Etiology, Diagnosis, and Treatment-A Literature Review. Int. J. Environ. Res. Public Health.

[4] Lobbezoo F., Ahlberg J., Raphael K.G., Wetselaar P., Glaros A.G., Kato T., Santiago V., Winocur E., De Laat A., De Leeuw R. (2018). International consensus on the assessment of bruxism: Report of a work in progress. J. Oral Rehabil..

[5] Mortazavi N., Tabatabaei A.H., Mohammadi M., Rajabi A. (2023). Is bruxism associated with temporomandibular joint disorders? A systematic review and meta-analysis. Evid. Based Dent..

[6] Zieliński G., Pająk-Zielińska B., Pająk A., Wójcicki M., Litko-Rola M., Ginszt M. (2025). Global co-occurrence of bruxism and temporomandibular disorders: A meta-regression analysis. Dent. Med. Probl..

[7] Song J.Y. (2021). Implant complications in bruxism patients. J. Korean Assoc. Oral Maxillofac. Surg..

[8] Thomas D.C., Manfredini D., Patel J., George A., Chanamolu B., Pitchumani P.K., Sangalli L. (2024). Sleep bruxism: The past, the present, and the future-evolution of a concept. J. Am. Dent. Assoc..

[9] Matusz K., Maciejewska-Szaniec Z., Gredes T., Pobudek-Radzikowska M., Glapiński M., Górna N., Przystańska A. (2022). Common therapeutic approaches in sleep and awake bruxism-an overview. Neurol. Neurochir. Pol..

[10] Casazza E., Giraudeau A., Payet A., Orthlieb J.D., Camoin A. (2022). Management of idiopathic sleep bruxism in children and adolescents: A systematic review of the literature. Arch. Pediatr..

[11] Kuang B., Li D., Lobbezoo F., de Vries R., Hilgevoord A., de Vries N., Huynh N., Lavigne G., Aarab G. (2022). Associations between sleep bruxism and other sleep-related disorders in adults: A systematic review. Sleep. Med..

[12] Chung J., Lobbezoo F., van Selms M.K.A., Chattrattrai T., Aarab G., Mitrirattanakul S. (2021). Physical, psychological and socio-demographic predictors related to patients’ self-belief of their temporomandibular disorders’ aetiology. J. Oral Rehabil..

[13] Caetano J.P., Goettems M.L., Nascimento G.G., Jansen K., da Silva R.A., Svensson P., Boscato N. (2024). Influence of malocclusion on sleep bruxism and orofacial pain: Data from a study in school children. Clin. Oral Investig..

[14] Kato T., Higashiyama M., Katagiri A., Toyoda H., Yamada M., Minota N., Katsura-Fuchihata S., Zhu Y. (2023). Understanding the pathophysiology of sleep bruxism based on human and animal studies: A narrative review. J. Oral Biosci..

[15] Smardz J., Martynowicz H., Wojakowska A., Michalek-Zrabkowska M., Mazur G., Wieckiewicz M. (2019). Correlation between Sleep Bruxism, Stress, and Depression-A Polysomnographic Study. J. Clin. Med..

[16] Firmani M., Reyes M., Becerra N., Flores G., Weitzman M., Espinosa P. (2015). Sleep bruxism in children and adolescents. Rev. Chil. Pediatr..

[17] Lobbezoo F., Ahlberg J., Manfredini D., Winocur E. (2012). Are bruxism and the bite causally related?. J. Oral Rehabil..

[18] Zieliński G., Pająk A., Wójcicki M. (2024). Global Prevalence of Sleep Bruxism and Awake Bruxism in Pediatric and Adult Populations: A Systematic Review and Meta-Analysis. J. Clin. Med..

[19] Cuschieri S. (2019). The STROBE guidelines. Saudi J. Anaesth..

[20] World Medical Association (2025). World Medical Association Declaration of Helsinki: Ethical Principles for Medical Research Involving Human Participants. JAMA..

[21] Klasser G.D., Rei N., Lavigne G.J. (2015). Sleep bruxism etiology: The evolution of a changing paradigm. J. Can. Dent. Assoc..

[22] Raja H.Z., Saleem M.N., Mumtaz M., Tahir F., Iqbal M.U., Naeem A. (2024). Diagnosis of Bruxism in Adults: A Systematic Review. J. Coll. Physicians Surg. Pak..

[23] Kanclerska J., Wieckiewicz M., Poreba R., Szymanska-Chabowska A., Gac P., Wojakowska A., Frosztega W., Michalek-Zrabkowska M., Mazur G., Martynowicz H. (2022). Polysomnographic Evaluation of Sleep Bruxism Intensity and Sleep Architecture in Nonapneic Hypertensives: A Prospective, Observational Study. J. Clin. Med..

[24] Howell M., Avidan A.Y., Foldvary-Schaefer N., Malkani R.G., During E.H., Roland J.P., McCarter S.J., Zak R.S., Carandang G., Kazmi U. (2023). Management of REM sleep behavior disorder: An American Academy of Sleep Medicine clinical practice guideline. J. Clin. Sleep. Med..

[25] Jeong M.Y., Kim M.J., Lim Y.J., Kwon H.B. (2024). Evaluation of eccentric tooth contact on a semi-adjustable articulator by using an occlusal analysis system. J. Prosthet. Dent..

[26] Li L., Chen H., Zhao Y., Wang Y., Sun Y. (2022). Design of occlusal wear facets of fixed dental prostheses driven by personalized mandibular movement. J. Prosthet. Dent..

[27] Pandey R., Kamble R. (2024). Comparative evaluation and co-relation in variation of curve of Spee and curve of Wilson in Class II div. 1, Class II div. 2, and Class III as against Class I malocclusion in central India population- an in vitro study. F1000Research.

[28] Dindaroğlu F., Duran G.S., Tekeli A., Görgülü S., Doğan S. (2016). Evaluation of the Relationship between Curve of Spee, WALA-FA Distance and Curve of Wilson in Normal Occlusion. Turk. J. Orthod..

[29] Aldayel A.M., AlGahnem Z.J., Alrashidi I.S., Nunu D.Y., Alzahrani A.M., Alburaidi W.S., Alanazi F., Alamari A.S., Alotaibi R.M. (2023). Orthodontics and Temporomandibular Disorders: An Overview. Cureus.

[30] Palumbo S.A., Robishaw J.D., Krasnoff J., Hennekens C.H. (2021). Different biases in meta-analyses of case-control and cohort studies: An example from genomics and precision medicine. Ann. Epidemiol..

[31] Serdar C.C., Cihan M., Yücel D., Serdar M.A. (2021). Sample size, power and effect size revisited: Simplified and practical approaches in pre-clinical, clinical and laboratory studies. Biochem. Med..

[32] Cadar M., Almăşan O. (2024). Dental occlusion characteristics in subjects with bruxism. Med. Pharm. Rep..

[33] Zieliński G., Gawda P. (2025). Defining Effect Size Standards in Temporomandibular Joint and Masticatory Muscle Research. Med. Sci. Monit..

[34] McHugh M.L. (2013). The chi-square test of independence. Biochem. Med..

[35] Iturriaga V., Bornhardt T., Velasquez N. (2023). Temporomandibular Joint: Review of Anatomy and Clinical Implications. Dent. Clin. North. Am..

[36] Xiaojie X., Yiling C., Honglei L., Jiamei P., Xiaoyong W., Hao Y., Hui C. (2024). Comparative analysis of myoelectric activity and mandibular movement in healthy and nonpainful articular temporomandibular disorder subjects. Clin. Oral Investig..

[37] Romero-Reyes M., Bassiur J.P. (2024). Temporomandibular Disorders, Bruxism and Headaches. Neurol. Clin..

[38] Poluha R.L., Canales G.T., Bonjardim L.R., Conti P.C.R. (2021). Oral behaviors, bruxism, malocclusion and painful temporomandibular joint clicking: Is there an association?. Braz. Oral Res..

[39] Grossi M.L., Castillo L.O., Pattussi M.P., Pinto G.M., Filho R.T. (2024). Validity between signs and symptoms of sleep bruxism against a validated portable electromyographic device. J. Clin. Exp. Dent..

[40] Manfredini D., Lobbezoo F. (2010). Relationship between bruxism and temporomandibular disorders: A systematic review of literature from 1998 to 2008. Oral Surg. Oral Med. Oral Pathol. Oral Radiol. Endod..

[41] Nagamatsu S.C., Minakuchi H., Clark G.T., Kuboki T. (2008). Relationship between the frequency of sleep bruxism and the prevalence of signs and symptoms of temporomandibular disorders in an adolescent population. Int. J. Prosthodont..

[42] Ita M.E., Ghimire P., Granquist E.J., Winkelstein B.A. (2022). MMPs in tissues retrieved during surgery from patients with TMJ disorders relate to pain more than to radiological damage score. J. Orthop. Res..

[43] Kluskens T.J., Kessler P.A., Jansma B.M., Kaas A., van de Ven V. (2023). Neural Correlates of Tooth Clenching in Patients with Bruxism and Temporomandibular Disorder-Related Pain. J. Oral Facial Pain Headache.

[44] Teng H., Sun T., Shu J., Shao B., Liu Z. (2025). Effect of Various Degrees of Anterior Disc Displacement on the Biomechanical Response of the Masticatory System. J. Biomech. Eng..

[45] Zhao Y.J., Chen J., Liu Y., Pan L.L., Guo Y.X., Zhang Z.M., Li Q., Chen Y.J. (2024). Regulation of CeA-Vme projection in masseter hyperactivity caused by restraint stress. Front. Cell Neurosci..

[46] Bornhardt T., Iturriaga V. (2021). Sleep Bruxism: An Integrated Clinical View. Sleep. Med. Clin..

[47] Tago C., Aoki S., Sato S. (2017). Status of occlusal contact during sleep bruxism in patients who visited dental clinics-A study using a Bruxchecke. Cranio.

[48] Fuentes A.D., Sforza C., Miralles R., Ferreira C.L., Mapelli A., Lodetti G., Martin C. (2017). Assessment of electromyographic activity in patients with temporomandibular disorders and natural mediotrusive occlusal contact during chewing and tooth grinding. Cranio.

[49] Mendoza-Martiarena Y., Paredes-Coz G., Alvarado-Menacho S., Watanabe-Velásquez R. (2025). Relationship between Mediotrusive Occlusal Contacts and Temporomandibular Disorders in Young Adults without Psychosocial Disorders: A Case-Control Study. J. Int. Soc. Prev. Community Dent..

[50] Klitynska O.V., Martyts Y.M., Gurando V.R., Layoch N.V. (2024). Effectiveness of bruxism treatment in young adults. Wiad. Lek..

[51] Nascimento B.L., Vieira A.R., Bezamat M., Ignácio S.A., Souza E.M. (2022). Occlusal problems, mental health issues and non-carious cervical lesions. Odontology.

[52] Oppitz L.R., Arantes A.C.M., Garanhani R.R., Costa C.A., Araujo C.M., Tanaka O.M., Andreis P.K.D.S., Schappo C., Ignácio S.A., Johann A.C.B.R. (2024). Efficiency of mixed and rigid occlusal stabilization splints: Randomized clinical trial. Braz. Oral Res..

[53] Albagieh H., Alomran I., Binakresh A., Alhatarisha N., Almeteb M., Khalaf Y., Alqublan A., Alqahatany M. (2023). Occlusal splints-types and effectiveness in temporomandibular disorder management. Saudi Dent. J..

[54] Ikeda K., Yamashita S. (2022). A Study for Determining the Inclination of the Occlusal Plane from the Mandibular Functional Trajectory. Int. J. Dent..

[55] Martin-Romanillos E., Feijóo G., Martín-Vacas A., Mourelle-Martínez M.R., Gallardo-López N.E., Caleya A.M. (2025). Analysis of the Relationship Between Unilateral Posterior Crossbite and Alterations in the Eruptive Trajectory of Maxillary Canines, the Occlusal Plane, and the Inclination of the Labial Commissure. Children.

[56] Myllymäki E., Heikinheimo K., Suominen A., Evälahti M., Michelotti A., Svedström-Oristo A.L., Rice D.P. (2023). Longitudinal trends in temporomandibular joint disorder symptoms, the impact of malocclusion and orthodontic treatment: A 20-year prospective study. J. Oral Rehabil..

[57] Yap A.U., Chen C., Wong H.C., Yow M., Tan E. (2021). Temporomandibular disorders in prospective orthodontic patients. Angle Orthod..

[58] Kapagiannidou D., Koutris M., Wetselaar P., Visscher C.M., van der Zaag J., Lobbezoo F. (2021). Association between polysomnographic parameters of sleep bruxism and attrition-type tooth wear. J. Oral Rehabil..

[59] Manfredini D., Ahlberg J., Lobbezoo F. (2022). Bruxism definition: Past, present, and future-What should a prosthodontist know?. J. Prosthet. Dent..

[60] Goldstein G., DeSantis L., Goodacre C. (2021). Bruxism: Best Evidence Consensus Statement. J. Prosthodont..

