# Supplementary material for: Sleep Bruxism and Occlusal Function: A Case–Control Study Based on Polysomnography in Young Colombians
Source: J Clin Med. 2025 Sep 24;14(19):6733. doi: 10.3390/jcm14196733 (PMC12524900; doi:10.3390/jcm14196733)
Supplement: Supplementary file 1 [file jcm-14-06733-s001.zip › jcm-3707942-supplementary.pdf]

STROBE Statement—checklist of items that should be included in reports of observational studies

	Item No.	Recommendation	Page No.	Relevant text from manuscript
**Title and abstract**	1	(*a*) Indicate the study's design with a commonly used term in the title or the abstract	1	
		(*b*) Provide in the abstract an informative and balanced summary of what was done and what was found	1	
		**Introduction**		
Background/rationale	2	Explain the scientific background and rationale for the investigation being reported	2	
Objectives	3	State specific objectives, including any prespecified hypotheses	3	
		**Methods**		
Study design	4	Present key elements of study design early in the paper	3	
Setting	5	Describe the setting, locations, and relevant dates, including periods of recruitment, exposure, follow-up, and data collection	3	
Participants	6	(*a*) *Cross-sectional study*—Give the eligibility criteria, and the sources and methods of selection of participants	3	
Variables	7	Clearly define all outcomes, exposures, predictors, potential confounders, and effect modifiers. Give diagnostic criteria, if applicable	3, 4	
Data sources/ measurement	8*	For each variable of interest, give sources of data and details of methods of assessment (measurement). Describe comparability of assessment methods if there is more than one group		
Bias	9	Describe any efforts to address potential sources of bias	4	
Study size	10	Explain how the study size was arrived at	4	

Continued on next page

Quantitative variables	11	Explain how quantitative variables were handled in the analyses. If applicable, describe which groupings were chosen and why	4
Statistical methods	12	(*a*) Describe all statistical methods, including those used to control for confounding	5
		(*b*) Describe any methods used to examine subgroups and interactions	5
		(*c*) Explain how missing data were addressed	5
		(*d*) *Cross-sectional study*—If applicable, describe analytical methods taking account of sampling strategy	5
		(*e*) Describe any sensitivity analyses	5
**Results**			
Participants	13*	(a) Report numbers of individuals at each stage of study—eg numbers potentially eligible, examined for eligibility, confirmed eligible, included in the study, completing follow-up, and analysed	5
		(b) Give reasons for non-participation at each stage	-
		(c) Consider use of a flow diagram	-
Descriptive data	14*	(a) Give characteristics of study participants (eg demographic, clinical, social) and information on exposures and potential confounders	5
		(b) Indicate number of participants with missing data for each variable of interest	5
		Cross-sectional study—Report numbers of outcome events or summary measures	*6,7*
Main results	16	(*a*) Give unadjusted estimates and, if applicable, confounder-adjusted estimates and their precision (eg, 95% confidence interval). Make clear which confounders were adjusted for and why they were included	6,7
		(*b*) Report category boundaries when continuous variables were categorized	6,7
		(*c*) If relevant, consider translating estimates of relative risk into absolute risk for a meaningful time period	-

Continued on next page

Other analyses	17	Report other analyses done—eg analyses of subgroups and interactions, and sensitivity analyses	6,7
**Discussion**			
Key results	18	Summarise key results with reference to study objectives	7
Limitations	19	Discuss limitations of the study, taking into account sources of potential bias or imprecision. Discuss both direction and magnitude of any potential bias	8,9
Interpretation	20	Give a cautious overall interpretation of results considering objectives, limitations, multiplicity of analyses, results from similar studies, and other relevant evidence	8,9
Generalisability	21	Discuss the generalisability (external validity) of the study results	8,9
**Other information**			
Funding	22	Give the source of funding and the role of the funders for the present study and, if applicable, for the original study on which the present article is based	10

*Give information separately for cases and controls in case-control studies and, if applicable, for exposed and unexposed groups in cohort and cross-sectional studies.

Note: An Explanation and Elaboration article discusses each checklist item and gives methodological background and published examples of transparent reporting. The STROBE checklist is best used in conjunction with this article (freely available on the Web sites of PLoS Medicine at http://www.plosmedicine.org/, Annals of Internal Medicine at http://www.annals.org/, and Epidemiology at http://www.epidem.com/). Information on the STROBE Initiative is available at www.strobe-statement.org.

Supplementary Materials S1: Self-report and intraoral clinical evaluation instrument

Participant Identification

Full Name:		
Age:		
Email:		
Phone Number:		
History of Orthodontic Treatment:	Yes:	
	No:	

Section I. *Risk Factors for Bruxism (Adapted from Kato et al., 2010)*

Please indicate whether the following conditions apply to you:			
1. ¿Do you smoke daily?	Yes:	No:	
2. ¿Do you consume alcohol (including beer) more than twice per week?	Yes:	No:	
3. ¿Do you drink more than 4 cups of coffee per day	Yes:	No:	
4. ¿Do you consider yourself to have a nervous or stressed personality?	Yes:	No:	
5. ¿Do you take medication for anxiety or depression?	Yes:	No:	
6. ¿Do you take sleep medication?	Yes:	No:	
7. ¿Do your parents or siblings grind their teeth (bruxism)?	Yes:	No:	Don't know:
8. ¿Do you know if you had bruxism during childhood?	Yes:	No:	Don't know:

Section II. *Self-Reported Sleep Bruxism Symptoms (Based on Klasser et al., 2015; Koyano et al., 2008)*

Please indicate whether the following conditions apply to you:		
1. ¿Has anyone heard you grinding your teeth at night?	Yes:	No:
2. ¿Do you experience jaw fatigue or pain when you wake up?	Yes:	No:
3. ¿Do your teeth or gums feel sore upon awakening?	Yes:	No:
4. ¿Do you experience headaches in the temple region upon waking?	Yes:	No
5. ¿Are you aware of grinding your teeth:		
¿During the day?	Yes:	No:
¿At night?	Yes:	No:
6. Are you aware of clenching your teeth:		
¿During the day?	Yes:	No:
¿At night?	Yes:	No:

Section III. *Clinical and Anamnestic Indicators of Bruxism (Based on Klasser et al., 2015; Koyano et al., 2008)*

1. Presence of wear facets consistent with mandibular function (working, balancing, protrusive) or in eccentric positions (bruxofacets)	Yes:	No:
2. Masseter muscle hypertrophy upon voluntary contraction	Yes:	No:
3. Complaint of masticatory muscle discomfort, fatigue or stiffness upon awakening	Yes:	No:
4. Tooth hypersensitivity to air or cold liquids	Yes:	No
5. Clicking or locking of the temporomandibular joint (TMJ)	Yes:	No:
6. Indentations on:		
Cheeks	Yes:	No:
Tongue	Yes:	No:

Section IV. *Diagnostic Criteria for Sleep Bruxism (Based on consensus guidelines: Koyano et al., 2008)*

1. ¿The patient reports or is aware of grinding or clenching sounds during sleep?	Yes:	No:
2. One or more of the following signs are present:		
Abnormal tooth wear (any location)	Yes:	No:
3. Discomfort, fatigue, or pain in jaw-related muscles upon awakening	Yes:	No:
4. TMJ pain or locking upon awakening	Yes:	No:
5. Masseter muscle hypertrophy after maximal voluntary clenching	Yes:	No:

Note: Muscular activity is not explained by another sleep disorder, medical or neurological condition, medication, or substance use.

Section V. *Individual Tooth Wear Index for Sleep Bruxism (Adapted for epidemiological use based on Koyano et al., 2008)*

**Score**	**Criteria**
0	No occlusal or incisal wear
1	Enamel wear with minimal shape loss; dentin not exposed
2	Dentin exposure affecting up to one-third of the tooth surface
3	Extensive dentin exposure, with more than one-third of crown height affected (includes restorations)

Tooth Wear Index

$$\text{Average Tooth Wear Index} = \frac{\sum_{i=1}^{n} P_i}{n}$$

Where:

Pi = individual wear score for tooth i (ranging from 0 to 3)

n = total number of teeth evaluated
